# The mobile seniors’ clinic - an innovative transition of care for frail older adults

**DOI:** 10.1186/s12877-024-05490-4

**Published:** 2024-11-05

**Authors:** Valérie Boucher, Eva-Marie Jouhair, Marie-Josée Sirois, Luc Tailleur, Philippe Voyer, Éric Mercier, Anik Giguère, Clermont E. Dionne, France Légaré, Clémence Dallaire, Stéphane Bergeron, Pierre-Hugues Carmichael, Marcel Emond

**Affiliations:** 1 CHU de Québec-Université Laval Research Centre, 1401 18ième rue, Québec, G1J1Z4 Canada; 2 Sorbonne Université, Paris, France; 3 CHU Lariboisière, Assistance Publique-Hôpitaux de Paris, Paris, France; 4 Faculté de médecine, Université Laval, Québec, Canada; 5 Centre intégré universitaire de santé et services sociaux (CIUSSS) de la Capitale-Nationale, Québec, Canada; 6 VITAM- Centre de recherche en santé durable, Québec, Canada; 7 Centre d’excellence sur le vieillissement de Québec (CEVQ), Québec, Canada

**Keywords:** Older adults, Aged, Hospital-at-home, Emergency department

## Abstract

**Background:**

This study aims to evaluate the impact of Quebec’s first hospital-at-home-inspired mobile Seniors’ Clinic, the “Clinique des Ainés (CDA)”, on frail older adults’ returns to the Emergency Department (ED), mortality, and hospital Length Of Stay (LOS) and rehospitalizations.

**Methods:**

*Design*: Quasi-experimental pre-post implementation cohort study. *Population*: Patients aged ≥ 75 years admitted to the short-term geriatric unit after an ED consultation (control) or included by the CDA (intervention). *Outcomes*: return to ED (RtoED), mortality, ED & hospital LOS, and rehospitalizations. *Statistical analyses*: Multivariable regression modelling.

**Results:**

Overall, 891 patients were included. At the intervention site (CDA) (*n* = 437), RtoED were similar at 30 (17.5% & 19.5%, *p* = 0.58), 90 (34.4% & 37.3%, *p* = 0.46) and 180 days (47.2% & 54.0%, *p* = 0.07) in the pre and post-implementation phases. No mortality differences were found. The hospitalization LOS was significantly shorter (28.26 and 14.22 days, *p* < 0.01). At 90 days, rehospitalization LOS was decreased by 8.51 days (*p* = 0.02) and by 6.48 days at 180 days (*p* = 0.03). Compared to the control site (*n* = 454) in the post-implementation phase, RtoED was 54% at the intervention site compared to 44.1% (*p* = 0.02) at 180 days. The CDA had a lower adjusted probability of mortality at 90 days compared to the control site (4.8% VS 11.7%, *p* = 0.03). No rehospitalization LOS differences were noted.

**Conclusions:**

The *Clinique des Ainés* showed effectiveness in caring for frail older patients in their homes by decreasing their hospital LOS by half and 90 days mortality risk. It was a safe care trajectory without a clinically significant increase in ED returns or mortality.

**Supplementary Information:**

The online version contains supplementary material available at 10.1186/s12877-024-05490-4.

## Background

The busy and overcrowded environment of most Emergency departments (EDs) is not well-suited for older adults [1, 2], who generally perceive being cared for in the ED as a distressing experience [3]. Several recommendations and guidelines have been published to improve the senior-friendliness of this environment [1, 4]. These guidelines state, among other things, that older ED patients’ length of stay (LOS) should not exceed eight hours [3, 5]. However, patients aged 75 years and over are more likely to experience prolonged ED LOS [6], possibly due to their more complex needs [7–9]. Increased ED LOS could have severe consequences for these patients, including delirium, painful procedures, experiencing functional decline, developing an acute condition, extended hospital stays, frequent returns to the ED, intensive care unit admission, and even transfer to long-term care facilities [6, 10–15]. Older patients often return to the ED within 30 days, and many are subsequently hospitalized [16, 17]. These repeated ED consultations contribute to overcrowding, which may impact the quality of care and healthcare expenditures [3, 18].

As older adults account for an ever-increasing proportion of ED and hospitalized patients [3, 19, 20], it has become clear that the ED environment, processes and care pathways must undergo a major transformation to meet the needs of these patients [7–9]. As hospitalization may negatively impact this age group [21–27], several innovative acute care pathways have been suggested for older patients: geriatric trauma units or ED, fast-track admissions, and systematic, comprehensive geriatric assessments before patient discharge. These new pathways can improve patient and caregiver satisfaction, although data regarding their impact on ED returns, LOS and rehospitalizations are scant [28–34].

The “hospital-at-home” concept, where hospital care is provided at patients’ homes, has emerged in many countries in response to the increasing demands for hospitalization [35] and its potentially harmful effects on older patients [36]. A systematic review of the impact of the hospital-at-home reports a decrease in six-month mortality and an improvement in functional abilities at 3, 6 and 12 months [35]. The hospital-at-home concept is also associated with reduced mortality, hospital readmissions, and costs with increased patient and employee satisfaction [37]. More recently, the feasibility of a hospital-at-home system was confirmed in the New York City area, where more than half of the patients were over 70 years old [38]. However, there is little evidence of the feasibility and relevance of this type of care pathway in a Canadian setting.

The first “hospital-at-home”-inspired geriatric clinic, the “Clinique des Ainés (CDA),” was created in 2018 in Quebec City, Canada. The primary objective of this study was to assess this CDA’s impact on decreasing patient return to the ED and mortality. The secondary objective was to evaluate the clinic’s effectiveness in decreasing ED and hospital Length of Stay and re-admissions to the hospital within 180 days.

## Methods

### Study design and setting

We used a two-site quasi-experimental design to assess the newly implemented CDA. Intervention site: The Hôpital Saint-François d’Assise, a university-affiliated hospital in the eastern part of Quebec City, Canada, replaced its short-term geriatric hospitalization unit by the CDA care pathway. The CDA facilitated the early discharge of frail older patients, who were provided at-home follow-ups by a multidisciplinary team of healthcare professionals. The team included physicians, nurses, nursing assistants, orderlies, occupational therapists, physiotherapists, nutritionists, pharmacists and social workers. The medical team was available 7 days a week between 8AM and 8PM, and the rehabilitation team and pharmacists were available between 8AM and 5PM. Although the services were provided at the patient’s home, the episode of care was classified as hospital care, with the CDA physician functioning as the attending physician. At the end of the CDA care, a summary of the episode of care was sent to the patient’s family physician, if they had one. Most CDA patients started physiotherapy during their hospital stay and continued treatment at home. IV fluids/ID antibiotics, as well as IV diuretics (Lasix) were provided in the home when needed by patients. A bladder scan was also at the disposal of the mobile team, however, this was the only diagnostic procedure available. The CDA team mainly relied on specific service trajectories with the hospital’s diagnostic services when necessary. The hospital’s short-term geriatric hospitalization unit (standard care pathway) and post-CDA care pathway are shown in Supplementary File 1.

Control site: We used a control standard care pathway site to capture temporal and systemic resource availability changes, which could impact our study outcome measures. The short-term geriatric hospitalization unit of the Institut de Cardiologie et de Pneumologie de Québec (IUCPQ) serves the western part of Quebec City and has an older patient population/ services/consultation profile similar to the CDA site. This hospital catchment area is also geographically distant enough to prevent inter-site contamination. The IUCPQ hospital did not implement any change to the short-term geriatric hospitalization unit during the study period.

The CHU de Québec-Université Laval Research Ethics Board, the Centre intégré universitaire de santé et de services sociaux de la Capitale-Nationale (CIUSSS-CN) Research Ethics Board, and the IUCPQ Research Ethics Board assessed this project. Per the Canadian Tri-Council Policy Statement, these three research ethics boards determined this study did not require ethics approval. Per the retrospective nature of this study, patient informed written consent was waived and waver is approved by the CHU de Québec-Université Laval Research Ethics Board. Our results are reported per the Transparent Reporting of Evaluations with Nonrandomized Designs (TREND) guidelines.

### Study population

This study included frail patients aged 75 years or older who met the following criteria: (1) suffering from at least one acute illness or decompensation of a chronic disease, (2) unavoidable hospitalization, (3) presenting an acute or sub-acute cognitive or functional decline, (4) having a potentially reversible condition requiring multidisciplinary intervention and (5) had a history of recurrent falls, delirium, major neurocognitive disorders, malnutrition/dehydration or pressure sores. Patients who died during their hospitalization or those admitted to a long-term care facility were excluded.

A medical archivist extracted a list of patients meeting these criteria and who were admitted to the Hôpital Saint-François d’Assise’s short-term geriatric unit (Intervention site) after an ED consultation between 2016 and 2018 (pre-implementation phase). Patients in the post-implementation phase (2019–2020) met the same criteria received the intervention and were therefore followed by the CDA team after a consultation in the ED. Inclusions in this group ended on December 1st, 2020, due to the CDA closure caused by the COVID-19 pandemic.

A list of patients meeting these same criteria was also extracted at the control site for the same periods (pre-implementation: 2016–2018, post-implementation: 2019–2020). A randomized selection was performed using the Statistical Analysis System (SAS Institute, Cary, NC, USA, v. 9.4).

### Data collection

A trained medical research archivist collected sociodemographic and clinical information from each ED consultation (index visit and returns within 180 days) from patients’ medical records using a standardized data collection form (Research electronic data capture (REDCap^®^). Information regarding patients’ pre-index visit functional status, comorbidities (Charlson Comorbidity Index [39], ED health services utilization, ED and hospital LOS, and discharge orientation were also collected.

### Outcomes measures

This study’s primary outcomes were patient returns to the ED and mortality at three specific time points- 30, 90, and 180 days. The central electronic medical record system enabled data collection on patients’ ED returns and deaths across the five primary university-affiliated hospitals in the Quebec City area.

The secondary outcome was the effectiveness in decreasing ED and hospital stay and readmissions. ED LOS was the time elapsed between triage and ED discharge. Hospital LOS was the number of days from admission to a hospital ward to discharge.

### Sample size

The sample size was estimated at 250 patients per phase at each site to detect a 45% between-group difference in the rate of ED returns at 90 days with an alpha = 0.05 and 80% power. This reduction was deemed necessary by the stakeholders associated with this project to ensure a notable decrease in the flow of older patients in the ED. Randomization and blinding were impossible in this study, as the decision to implement the CDA care pathway was made at an organizational level at only one site.

### Statistical analyses

Patients’ characteristics were compared using χ2 and Student t for categorical and continuous data, respectively. A propensity score was computed to adjust for systematic between-site differences on the following covariates: the Charlson Comorbidity Index score, prior autonomy in daily activities, sex, fall recurrence, delirium, cognitive impairment, malnutrition, dehydration, and pressure sores. Multivariable regression modelling was used to isolate and assess the impact of the intervention (CDA) on return to the ED, hospital readmission and LOS. The regression model included the propensity score, age, number of previous ED consultations and reasons for consultation. Statistical significance was set at *p* < 0.05. All statistical analyses were performed using the Statistical Analysis System (SAS Institute, Cary, NC, USA, v. 9.4).

## Results

### Characteristics of the study population

A total of 437 patients were included at the intervention site: 248 in the pre-implementation phase, and 189 were followed by the CDA (post-implementation). At the control site, 454 patients were included: 248 and 206 in the pre- and post-implementation phases. The mean age was 86.7 ± 6.0 and 87.5 ± 5.9, and women represented 72.3% and 65.0% at the intervention and control sites, respectively. Most of our patients lived in retirement homes (intervention site: 54.1%, control site: 50.2%) and were autonomous or needed partial assistance to perform their activities of daily living/instrumental activities of daily living. The mean Charlson Comobidity Index score was 4.3 ± 2.6 for the intervention site and 3.5 ± 2.4 for the control site. Table 1 shows the patients’ socio-demographic and clinical characteristics.

**Table 1:** Baseline characteristics of the study samples

	Intervention site (CDA) *n* = 437 *n* (%)	Control site *N* = 454 *n* (%)	*P* value
**Sociodemographic information**			
Age, mean ± SD	86.7 ± 6.0	87.5 ± 5.9	0.06
Age, median [IQR]	87.2 [82.7–90.7]	87.6 [83.4–92.0]	
≥ 85	270 (62.5)	291 (64.5)	0.53
Sex, Female	316 (72.3)	295 (65.0)	**0.02**
Living situation			**0.01**
Home alone, without help*	63 (14.6)	87 (19.2)	
Home alone, with help*	9 (2.1)	2 (0.4)	
Home with family members	74 (17.1)	102 (22.5)	
Home with family and help*	13 (3.0)	12 (2.6)	
Retirement homes	235 (54.1)	228 (50.2)	
Long-term care	39 (9.0)	23 (5.1)	
**Patient history**			
Charlson Comorbidity Index score			
Mean ± SD	4.3 ± 2.6	3.5 ± 2.4	**< 0.01**
0	14 (3.2)	30 (6.6)	**< 0.01**
1–2	112 (25.6)	148 (32.6)	
3–4	119 (27.2)	137 (30.2)	
≥ 5	192 (43.9)	139 (30.6)	
Comorbidities			
Cognitive impairment	267 (61.1)	142 (31.3)	**< 0.01**
Hypertension	336 (76.0)	382 (84.1)	**0.01**
Alcohol use disorder	44 (10.1)	31 (6.8)	0.08
Heart rhythm disorder	144 (33.0)	239 (52.6)	**< 0.01**
Neurosensory disorder	264 (60.7)	153 (33.7)	**< 0.01**
Neurological gait disorder	84 (19.2)	24 (5.3)	**< 0.01**
**Pre-ED functional status**			
Activities of daily living			**< 0.01**
Autonomous	177 (41.0)	237 (53.3)	
Partial assistance	230 (53.2)	192 (43.2)	
Total assistance/dependent	25 (5.8)	16 (3.6)	
Instrumental activities of daily living			**< 0.01**
Autonomous	38 (8.9)	57 (12.9)	
Partial assistance	211 (49.2)	249 (56.2)	
Total assistance/dependent	180 (42.0)	137 (30.9)	
**Health services utilization**			
≥ 1 ED visit 6 months before the index visit	226 (51.7)	235 (51.8)	0.99
Reason for index ED visit			
Fall	205 (46.9)	118 (26.0)	**< 0.01**
Decreased general condition	143 (32.7)	170 (37.4)	0.14
Pain	162 (37.1)	150 (33.0)	0.21
Confusion	119 (27.2)	71 (15.6)	**< 0.01**
Neurological symptoms	29 (6.6)	41 (9.0)	0.18
Dyspnea	28 (6.4)	48 (10.6)	**0.03**
Digestive symptoms	18 (4.1)	51 (11.2)	**< 0.01**
Syncope / lipothymia	16 (3.7)	26 (5.7)	0.15
Deconditioning	7 (1.6)	4 (0.9)	0.33
Urological symptoms	13 (3.0)	20 (4.4)	0.26
Other	51 (11.7)	100 (22.0)	**< 0.01**
ED patient disposition*			
Discharged home	52 (12.0)	0 (0.0)	-
Hospitalized	373 (85.4)	454 (100.0)	
Other	7 (1.6)	0 (0.0)	

CDA : Clinique des aînés

*help: private or publicly funded

Patients’ characteristics according to phase are shown by site in Table 2. At the intervention site (CDA), patients who received the standard care pathway (pre-implementation) included a larger proportion of females (*p* = 0.02), more patients with neurosensory disorder (*p* < 0.01), and fewer patients needing assistance in daily instrumental activities (*p* = 0.03). Reasons for ED index consultations were similar in the pre-and post-phases, except for confusion (*p* = 0.01) and deconditioning (*p* = 0.02).

**Table 2:** Patients’ characteristics in pre- and post-implementation phases, by site

	Intervention site (CDA)			Control site		
	Pre-Implementation phase *n* = 248 *n* (%)	Post-Implementation phase *n* = 189 *n* (%)	*P* value	Pre -Implementation phase *n* = 248 *n* (%)	Post-Implementation phase *n* = 206 *n* (%)	*P* value
**Sociodemographic information**						
Age, mean ± SD	86.4 ± 5.8	87.1 ± 6.4	0.28	87.7 ± 5.9	87.6 ± 5.6	0.45
≥ 85	149 (60.1)	121 (65.8)		163 (66.0)	128 (62.8)	
Sex, Female	190 (76.6)	126 (66.7)	**0.02**	169 (68.2)	126 (61.2)	0.12
Living situation						
Home alone, no help	41 (16.8)	22 (11.6)		40 (16.1)	47 (22.8)	
Home alone + help	7 (2.9)	2 (1.1)		2 (0.8)	0 (0.0)	
Home with family	34 (13.9)	40 (21.2)		52 (21.0)	50 (24.3)	
Home with family + help	7 (2.9)	6 (3.2)		9 (3.6)	3 (1.5)	
Retirement home	140 (57.4)	95 (50.3)		129 (52.0)	99 (48.1)	
Long-term care	15 (6.2)	24 (12.7)		16 (6.5)	7 (3.4)	
**Patient history**						
Charlson Comorbidity Index score						
Mean ± SD	4.3 ± 2.7	4.3 ± 2.5	0.97	3.6 ± 2.4	3.5 ± 2.5	0.77
0	9 (3.6)	5 (2.7)		16 (6.5)	14 (6.8)	
1–2	66 (26.6)	46 (24.3)		82 (22.1)	66 (32.0)	
3–4	64 (25.8)	55 (29.1)		71 (28.6)	66 (32.0)	
≥ 5	109 (44.0)	83 (43.9)		79 (31.9)	60 (29.1)	
Comorbidities						
Cognitive impairment	144 (58.1)	123 (65.1)	0.14	75 (30.2)	67 (32.5)	0.60
Hypertension	191 (77.0)	145 (76.7)	0.94	203 (81.9)	179 (86.9)	0.14
Alcohol use disorder	27 (10.9)	17 (9.0)	0.51	13 (5.2)	18 (8.7)	0.14
Heart rhythm disorder	89 (35.9)	55 (29.1)	0.13	133 (53.6)	106 (51.5)	0.64
Neurosensory disorder	164 (66.4)	100 (53.2)	**< 0.01**	88 (35.5)	65 (31.6)	0.38
Neurological gait disorder	49 (19.8)	35 (18.5)	0.74	21 (8.5)	3 (1.5)	**< 0.01**
**Pre-ED functional status**						
Activities of daily living			0.78			0.30
Independent	99 (40.2)	78 (42.0)		120 (50.0)	117 (57.1)	
Partial assistance	134 (54.5)	96 (51.6)		110 (45.8)	82 (40.0)	
Dependent	13 (5.3)	12 (6.5)		10 (4.2)	6 (2.9)	
Instrumental activities of daily living			**0.03**			0.24
Independent	22 (9.0)	16 (8.7)		27 (11.4)	30 (14.6)	
Partial assistance	133 (54.5)	78 (42.2)		129 (54.4)	120 (58.3)	
Dependent	89 (36.5)	91 (49.2)		81 (34.2)	56 (27.2)	
**Health services utilization**						
≥ 1 ED visit 6 months before the index visit	125 (50.4)	101 (53.4)	0.52	131 (52.8)	104 (50.5)	0.62
**Index ED visit**						
Reason for ED consultation						
Fall	121 (48.8)	84 (44.4)	0.37	61 (24.6)	57 (27.7)	0.46
Decreased general condition	90 (36.3)	53 (28.0)	0.07	76 (30.7)	94 (45.6)	**< 0.01**
Pain syndrome	83 (33.5)	79 (41.8)	0.07	81 (32.7)	69 (33.5)	0.85
Confusion	79 (31.9)	40 (21.2)	**0.01**	30 (12.1)	41 (19.9)	**0.02**
Neurological symptoms	17 (6.9)	12 (6.4)	0.83	19 (7.7)	22 (10.7)	0.26
Dyspnea	15 (6.1)	13 (6.9)	0.73	18 (7.3)	30 (14.6)	**0.01**
Digestive symptoms	11 (4.4)	7 (3.7)	0.70	21 (8.5)	30 (14.6)	**0.04**
Syncope / lipothymia	11 (4.4)	5 (2.7)	0.32	14 (5.7)	12 (5.8)	0.93
Deconditioning	7 (2.9)	0 (0.0)	**0.02**	4 (1.6)	0 (0.0)	0.07
Urological symptoms	6 (2.4)	7 (3.7)	0.43	5 (2.0)	15 (7.3)	**< 0.01**
Other	32 (12.9)	19 (10.1)	0.36	50 (20.2)	45 (21.8)	0.93
**ED patient disposition**			**< 0.01**			-
Discharged home	0 (0.0)	52 (28.3)		0 (0.0)	0 (0.0)	
Hospitalized	248 (100.0)	125 (67.9)		248 (100.0)	206 (100.0)	
Convalescence	0 (0.0)	5 (2.7)		0 (0.0)	0 (0.0)	
Relocation	0 (0.0)	2 (1.1)		0 (0.0)	0 (0.0)	

CDA: Clinique des aînés; SD: Standard Deviation ; IQR: Interquartile Range ; ED: Emergency Department

At the control site, patients were similar across phases except for neurological gait disorders, which were more prevalent in the pre-implementation phase (*p* < 0.01). The following reasons for index consultations were found in greater proportion in the post-implementation phase: decreased general condition (*p* = 0.001), confusion (*p* = 0.02), dyspnea (0.01), digestive symptoms (*p* = 0.04), and urological symptoms (*p* = 0.007).

### Returns to the ED

Table 3 shows the proportions and adjusted probability of returns to the ED at 30, 90 and 180 days. The adjusted probability of returning to the ED at 30, 90 and 180 days was similar between phases at both sites.

**Table 3:** Length of stays and probability of ED returns, rehospitalizations and deaths modelling*

	Intervention site (CDA)			Control site		
	*Pre-Implementation* *n* = 248 Mean [95% CI]	*Post-Implementation* *n* = 189 Mean [95% CI]	p-value	*Pre-Implementation* *n* = 248 Mean [95% CI]	*Post-Implementation* *n* = 206 Mean [95% CI]	p-value
**Index ED visit**						
Adjusted ED LOS, days	1.65 [1.42–1.91]	1.73 [1.49–2.01]	0.16	0.73 [0.61–0.87]	0.66 [0.55–0.79]	0.28
Adjusted hospital LOS, days	28.26 [22.57–35.38]	14.22 [10.80-18.73]	**< 0.01**	15.82 [12.39–20.20]	13.83 [10.89–17.56]	0.19
**30 days**						
***≥ 1 return to the ED***,*** n (%)***	51 (20.6)	40 (21.2)		72 (29.0)	54 (26.2)	
Adjusted probability, %	17.5 [10.5–29.1]	19.5 [11.5–32.8]	0.58	25.1 [15.2–41.5]	23.0 [14.5–36.2]	0.56
Adjusted ED LOS, days	0.94 [0.50–1.79]	0.98 [0.51–1.89]	0.80	0.56 [0.29–1.05]	0.50 [0.25–0.98]	0.58
***≥ 1 Rehospitalization***,*** n (%)***	23 (9.3)	31 (16.4)		44 (17.7)	37 (18.0)	
Adjusted probability, %	8.4 [4.1–17.2]	14.6 [7.3–29.2]	**0.03**	16.2 [8.2–31.6]	15.6 [8.4–28.9]	0.87
Adjusted hospital LOS, days	6.53 [1.22–34.79]	3.56 [0.34–29.28]	0.27	8.12 [1.76–37.48]	3.52 [0.58–21.53]	0.06
***Death***,*** n (%)***	5 (2.0)	2 (1.1)		10 (4.0)	10 (4.9)	
Adjusted probability, %	-	-	-	-	-	-
**90 days**						
***≥ 1 return to the ED***,*** n (%)***	106 (42.7)	83 (43.9)		125 (50.4)	86 (41.8)	
Adjusted probability, %	34.4 [24.7–47.9]	37.3 [26.4–52.6]	0.46	39.9 [28.4–56.1]	34.4 [24.9–47.4]	0.16
Adjusted ED LOS, days	1.59 [1.01–2.49]	1.47 [0.93–2.31]	0.52	0.94 [0.58–1.53]	1.19 [0.77–1.84]	0.15
***≥ 1 Rehospitalization***,*** n (%)***	58 (23.4)	55 (29.1)		87 (35.1)	60 (29.1)	
Adjusted probability, %	21.5 [13.5–34.2]	27.8 [17.4–44.3]	0.12	33.0 [21.2–51.5]	27.2 [17.8–41.6]	0.17
Adjusted hospital LOS, days	16.92 [9.22–31.04]	8.41 [3.88–18.22]	**0.02**	16.94 [9.29–30.87]	12.19 [6.40-23.21]	0.13
***Death***,*** n (%)***	14 (5.7)	10 (5.3)		27 (10.9)	22 (10.7)	
Adjusted probability, %	6.1 [2.1–17.2]	4.8 [1.6–14.9]	0.61	12.2 [4.6–32.1]	11.7 [4.8–28.7]	0.89
**180 days**						
***≥ 1 return to the ED***,*** n (%)***	147 (59.3)	118 (62.4)		159 (64.1)	109 (52.9)	
Adjusted probability, %	47.2 [37.2–59.9]	54.0 [42.4–68.7]	0.07	50.0 [39.0-64.1]	44.1 [34.6–56.1]	0.13
Adjusted ED LOS, days	1.11 [0.63–1.96]	1.01 [0.58–1.76]	0.47	0.71 [0.38–1.33]	0.91 [0.53–1.54]	0.19
***≥ 1 Rehospitalization***,*** n (%)***	89 (35.9)	90 (47.6)		124 (50.0)	80 (38.8)	
Adjusted probability, %	29.6 [20.8–42.2]	41.2 [29.2–58.1]	**0.01**	38.8 [27.6–54.6]	32.6 [23.4–45.5]	0.10
Adjusted hospital LOS, days	13.56 [7.29–25.19]	7.08 [3.26–15.37]	**0.03**	15.13 [8.19–27.96]	10.64 [5.62–20.12]	0.13
***Death***,*** n (%)***	22 (8.9)	19 (10.1)		44 (17.7)	33 (16.0)	
Adjusted probability, %	9.8 [4.7–20.3]	11.9 [5.7–24.8]	0.51	21.8 [11.2–42.2]	19.2 [10.4–35.4]	0.55

CDA: Clinique des aînés; CI: Confidence Interval; ED : Emergency Department; SD: Standard Deviation; LOS: Length Of Stay

*Adjusted for propensity score, age, number of previous consultations and reason for consultation

**Fig. 1:**
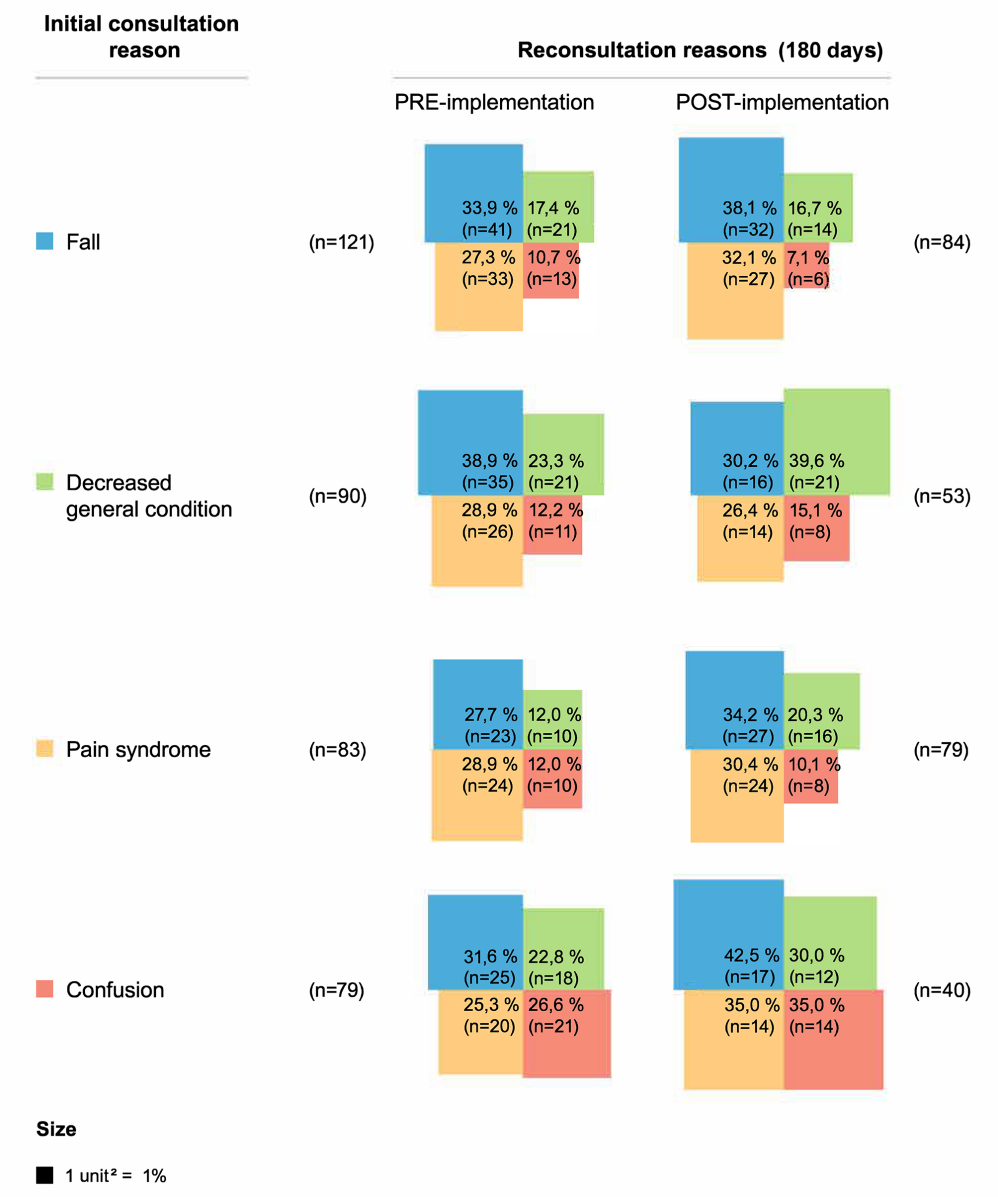
Intervention site (CDA): reasons for emergency department returns. CDA: Clinique des aînés

**Fig. 2:**
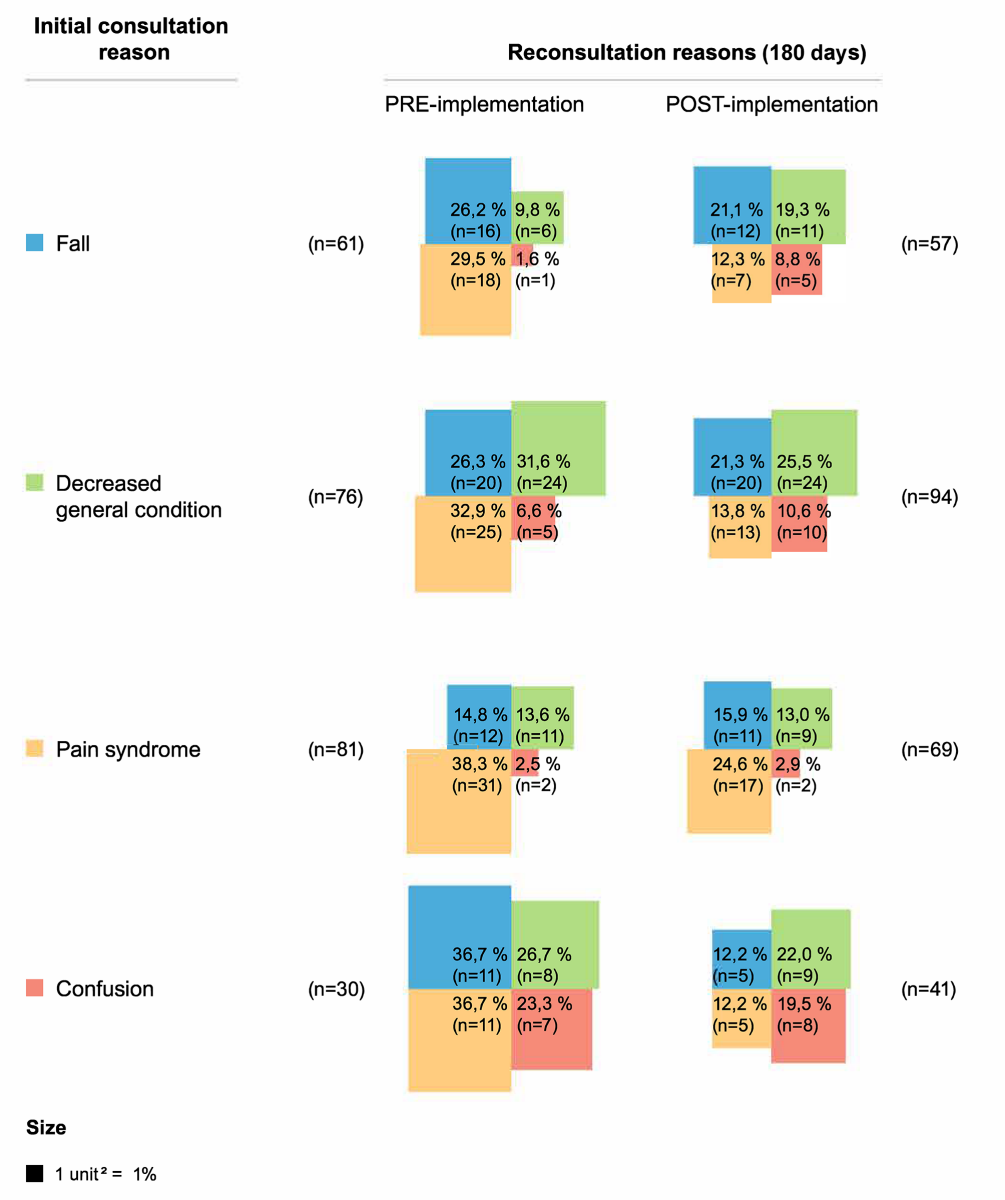
Control site: reasons for emergency department returns

The reasons for four of the most frequent ED returns are shown in Figs. 1 and 2 for both sites. At the intervention site (CDA), depending on the specific reason for admission, 23.3-56.1% and 30.4-59.0% of patients returned for the same problem in the pre-and post-implementation phases. Similarly, 23.3-68.1% returned for the same problem during the pre-implementation phase and 19.5-62.3% during the post-implementation phase at the control site.

When comparing the two sites (Supplemental material 2), we found that patients from the control site had a higher adjusted probability of returning to the ED at 30 days in the pre-implementation phase compared to the CDA site (25.1% VS 17.5%, *p* = 0.03). In the post-implementation phase, the adjusted probability of returning to the ED at 180 days was 54.0% for the CDA patients and 44.1% for patients from the control site (*p* = 0.02) (Supplemental material 2).

### Mortality

At 90 and 180 days, there were no pre/post-implementation differences pertaining to patient mortality at both sites. The small number of deaths at 30 days precluded statistical analyses (Table 3).

The CDA site had a lower adjusted probability of mortality at 90 days compared to the control site in both the pre- (6.1% VS 12.2%, *p* = 0.03) and post-implementation phases (4.8% VS 11.7%, *p* = 0.03). This difference is also observed in the pre-implementation phase at 180 days, with a lower adjusted probability of mortality in patients from the CDA (9.8% VS 21.8%, *p* < 0.01) (Supplemental material 2).

### LOS and hospital readmissions

#### ED and hospital LOS – initial visit

Table 3 shows the ED and Hospital LOS at the initial ED visit according to site and phase. Although there was no difference in ED LOS, the hospitalization LOS was significantly shorter in patients discharged with the CDA mobile services (28.26 and 14.22 days, *p* < 0.01) for the pre-and post-implementation phases, respectively. There were no differences in initial visit LOS between phases at the control site (ED LOS: *p* = 0.28, hospital LOS: *p* = 0.19).

Between-site comparisons show that patients from the CDA site had longer ED LOS than those from the control site during both phases (pre-implementation:1.65 days VS 0.73 days, *p* < 0.01; post-implementation: 1.73 days VS 0.66 days, *p* < 0.01). In the pre-implementation phase, they also had a significantly longer hospital LOS than the control site (28.26 days VS 15.82 days, *p* < 0.01). In the post-implementation phase, both sites’ hospital LOS were similar (Supplemental material 2).

#### ED return LOS

As shown in Table 3, ED return LOS were similar between phases at each site. Compared to the CDA site, the control site had significantly lower ED LOS during the pre-implementation phase at 30 (*p* < 0.01), 90 (*p* < 0.01) and 180 days (*p* = 0.01). During the post-implementation phase, the control site still had lower ED LOS at 30 days (*p* < 0.01), but similar LOS were recorded between sites at 90 and 180 days.

#### Rehospitalization and LOS

During the post-implementation phase, the adjusted probability of rehospitalization was higher for patients from the CDA site at 30 and 180 days, as compared to the pre-implementation phase (30 days: 14.6% VS 8.4%, *p* = 0.03; 180 days: 41.2% VS 29.6%, *p* = 0.01). However, this difference is not observed at 90. Patients from the control site had similar rehospitalization probabilities between phases at all time points (Table 3). A significant 8.51-day decrease in hospital LOS was found in patients from the post-implementation phase at the CDA site at 90 days (*p* = 0.02) and a 6.48 decrease at 180 days (*p* = 0.03). Hospital LOS were similar at all time points between phases at the control site.

During the pre-implementation phase, the CDA site had a lower adjusted probability of rehospitalization than the control site. This was seen both at 30 days (8.4% vs. 16.2%, *p* = 0.01), 90 days (21.5% vs. 33.0%, *p* < 0.01), and 180 days (29.6% vs. 38.8%, *p* = 0.01). In the post-implementation phase, patients from the CDA site had a greater adjusted probability of rehospitalization at 180 days (41.2% VS 32.6%, *p* = 0.04). The rehospitalization LOS was similar between the sites in both phases.

## Discussion

This study is the first to evaluate the effects of a hospital-at-home-inspired mobile clinic for frail older patients in a Canadian context. Although there was no difference in ED LOS during the patient’s initial consultation, the hospitalization LOS was 14.04 days shorter in patients discharged with the CDA mobile services. Rehospitalization LOS was consistently cut in half in patients whom the CDA followed, and this was statistically significant at 90 and 180 days. Based on our findings, the implementation of the CDA did not improve or negatively impact the probability of returning to the ED, which remained similar between phases. The probability of ED returns was also similar to those of the control site in the post-implementation phase at 30 and 90 days. Furthermore, implementing the CDA did not negatively impact the rates and adjusted probability of mortality.

Our findings indicate that the CDA did not negatively impact patient mortality and returns to the ED but significantly reduced hospital stay length. The very environment of the in-hospital units, where patients have no access to their personal belongings and are exposed to prolonged bed rest, drastic dietary changes, and the introduction of new medications, can lead to a decline in hospitalized older adults [21–24]. This loss of function can result in an inability to return home and significant costs [40, 41]. Therefore, the reduction in hospital LOS of CDA patients is an important finding. Although a recent Cochrane review found no difference in 6-month mortality [42], other authors have shown that the hospital-at-home concept may decrease patient mortality [35, 37]. However, our small number of deaths prevents us from drawing firm conclusions on the impact of the CDA on patient mortality.

Our results do not show a decrease in hospital readmissions or ED returns, as was found by some authors [37, 43]. The potential immaturity of a new system could account for this. The CDA was the first of its kind in the Province of Quebec, and despite being fully implemented, this extensive organizational change may not yield all immediate benefits. Additionally, it is possible that the CDA lacked solid communication channels and patient pathways with other resources. For example, a 911 call from a CDA patient for a non-life-threatening health problem could have been transferred to a CDA resource to avoid transport to an ED. A comprehensive knowledge translation and communication strategy was devised through the attached research project to ensure that the relevant information regarding the clinic was disseminated to all stakeholders. However, the Clinic’s activities were cut short due to the COVID-19 pandemic.

It is important to consider not only the organizational aspect but also the intrinsic characteristics of those older patients. On average, these patients were 86 years old, and a third of them had a Charlson Comorbidity Index score ≥ 5, which means they had a high one-year mortality rate [39, 44]. Patients treated at the CDA were discharged earlier and with increased risk-taking for returning home. Conversely, patients who received the pre-implantation care pathway and those from the control site who were discharged home were more carefully selected for a return home. Some riskier patient discharges may have been relocated and thus not included in the pre-implementation and control site cohorts. These patients are generally significant healthcare services users. Moreover, due to the pandemic, some post-implantation patients returned home with reduced homecare resources and COVID isolations, which was not ideal. Despite this, the CDA did not have any significant negative impact on the returns to the ED and rehospitalizations. Instead, it successfully and significantly reduced the hospital LOS of those patients, which is a substantial benefit for both patients and the healthcare system.

While being at home is reassuring, the new care process may unsettle those accustomed to traditional care. Although some authors found that patients receiving care at home reported better experiences compared to those who were hospitalized [45], future research should assess the acceptability of the hospital-at-home concept according to older Canadian patients, their caregivers and healthcare professionals. Further, it would be interesting to determine the impact of being cared for in their home on patient-centered outcomes, such as functional and cognitive functioning, compared to the standard care pathway. It would also be important to perform an economic assessment to quantify the impact of hospital-at-home care on healthcare expenditures. Some authors reported that the hospital-at-home concept was used to help alleviate the pandemic’s burden on the healthcare system [46]. It would have been interesting to assess the impact of the CDA in the COVID-19 context had it remained operational.

### Limitations

This study has limitations. First, its quasi-experimental nature may have introduced a selection bias in the CDA pre-implementation phase, as well as for control patients. The retrospective data collection may have skewed the patient’s functional status assessment. Furthermore, while we successfully obtained data on ED returns and rehospitalizations via the electronic medical record system (five sites), there is a possibility that visits to hospitals outside of the network may have been overlooked. This may have slightly underestimated the number of ED returns and patient rehospitalizations from both sites. However, since our population consists of frail older adults, we believe that rehospitalizations outside the regular regional catchment area are highly unlikely. Between-site differences in patients’ characteristics could also have influenced returns to ED, which is why our multivariable regression model included a propensity score to adjust for systematic between-site differences. Lastly, we could not reach our sample size due to the CDA closure due to the COVID-19 pandemic.

## Conclusion

The ‘‘Clinique des Ainés’’ showed effectiveness in caring for frail older people in their homes by decreasing the length of their hospital stay by half that of the standard short-term geriatric unit of the pre-implementation phase. It was also a safe care trajectory since it did not significantly increase ED returns and mortality. Our results confirm that a hospital-at-home-inspired mobile clinic aiming to reduce Emergency Department returns and length of stay requires significant organizational and societal changes. This can only be achieved through a well-structured process that involves an extensive knowledge translation plan and structured communication channels.

## Electronic supplementary material

Below is the link to the electronic supplementary material.

Supplementary Material 1

## Data Availability

The datasets used and/or analysed during the current study are available from the corresponding author on reasonable request.

## Abbreviations

CDA: Clinique des aînés
ED: Emergency Department
LOS: Length Of Stay
RtoED: Return to ED
IUCPQ: Institut de Cardiologie et de Pneumologie de Québec
CIUSSS-CN: Centre intégré universitaire de santé et de services sociaux de la Capitale-Nationale
TREND: Transparent Reporting of Evaluations with Nonrandomized Designs
