# Supplementary material for: The mobile seniors’ clinic - an innovative transition of care for frail older adults
Source: BMC Geriatr. 2024 Nov 5;24:914. doi: 10.1186/s12877-024-05490-4 (PMC11536935; doi:10.1186/s12877-024-05490-4)
Supplement: Supplementary file 1 — Supplementary Material 1 [file 12877_2024_5490_MOESM1_ESM.docx]

Supplemental Material 1. Care trajectories.

Pre-implementation and Control site care trajectory (2016-2017)


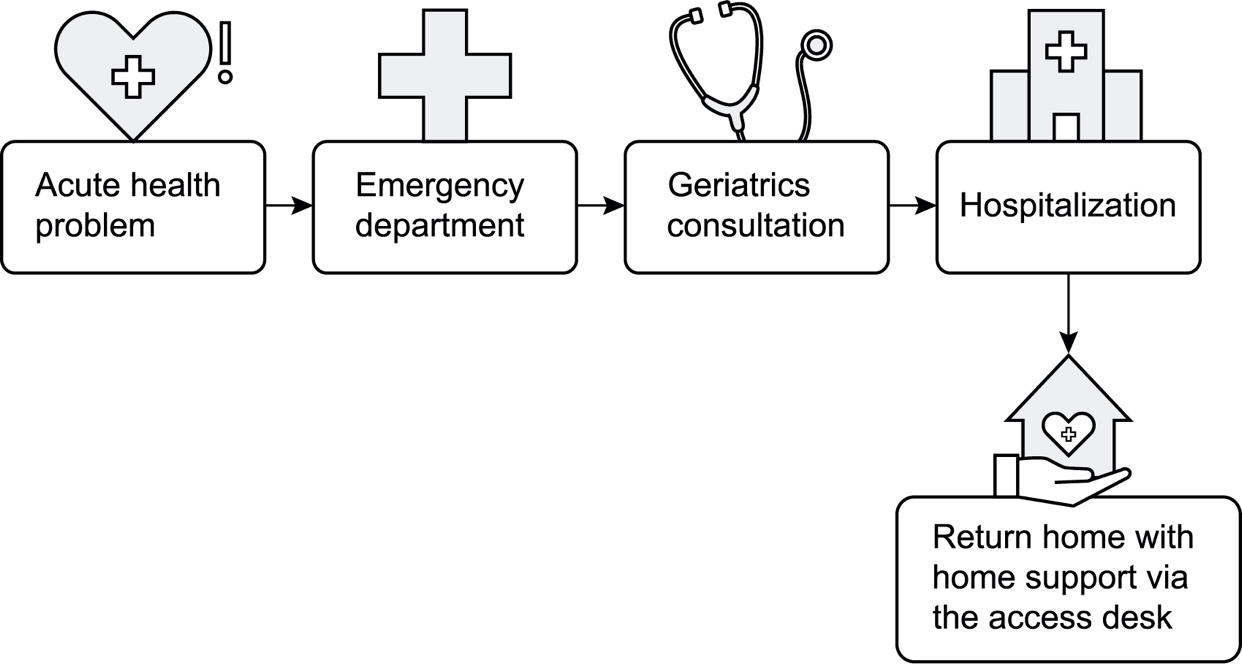


Post-implementation care trajectory at the Intervention site (CDA) (2019-2020)


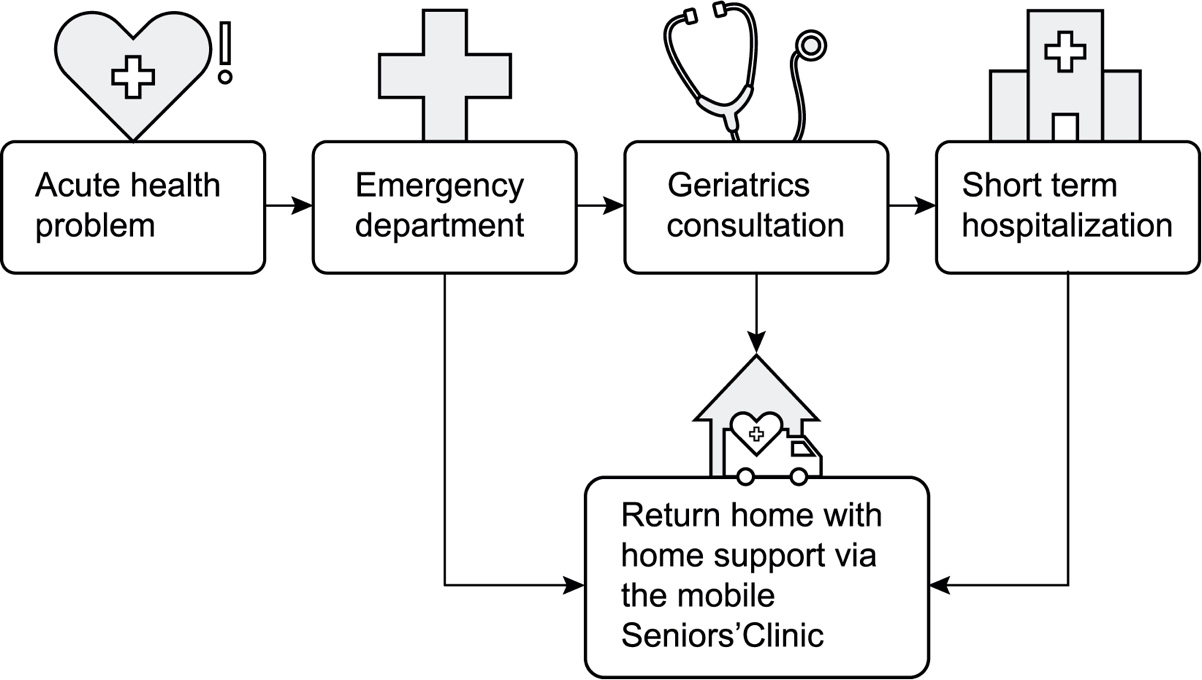


**Supplemental Material 2. Length of Stays, and probability of ED returns, Rehospitalizations and Deaths modelling* - Site Comparisons**

|  | **Pre-implementation** | | | **Post-implementation** | | |
| --- | --- | --- | --- | --- | --- | --- |
|  | *Intervention site (CDA)*  *n=248*  Mean [95% CI] | *Control site*  *n=248*  Mean [95% CI] | p-value | *Intervention site (CDA)*  *n=189*  Mean [95% CI] | *Control site*  *n=206*  Mean [95% CI] | p-value |
| **Index ED visit** |  |  |  |  |  |  |
| Adjusted ED LOS, days | 1.65 [1.42-1.91] | 0.73 [0.61-0.87] | **<0.01** | 1.73 [1.49-2.01] | 0.66 [0.55-0.79] | **<0.01** |
| Adjusted hospital LOS, days | 28.26 [22.57-35.38] | 15.82 [12.39-20.20] | **<0.01** | 14.22 [10.80-18.73] | 13.83 [10.89-17.56] | 0.82 |
| **30 days** |  |  |  |  |  |  |
| ***≥1 return to the ED, n (%)*** | 51 (20.6) | 72 (29.0) |  | 40 (21.2) | 54 (26.2) |  |
| Adjusted probability, % | 17.5 [10.5-29.1] | 25.1 [15.2-41.5] | **0.03** | 19.5 [11.5-32.8] | 23.0 [14.5-36.2] | 0.37 |
| Adjusted ED LOS, days | 0.94 [0.50-1.79] | 0.56 [0.29-1.05] | **<0.01** | 0.98 [0.51-1.89] | 0.50 [0.25-0.98] | **<0.01** |
| ***≥1 Rehospitalization, n (%)*** | 23 (9.3) | 44 (17.7) |  | 31 (16.4) | 37 (18.0) |  |
| Adjusted probability, % | 8.4 [4.1-17.2] | 16.2 [8.2-31.6] | **0.01** | 14.6 [7.3-29.2] | 15.6 [8.4-28.9] | 0.78 |
| Adjusted hospital LOS, days | 6.53 [1.22-34.79] | 8.12 [1.76-37.48] | 0.47 | 3.56 [0.34-29.28] | 3.52 [0.58-21.53] | 0.86 |
| ***Death, n (%)*** | 5 (2.0) | 10 (4.0) |  | 2 (1.1) | 10 (4.9) |  |
| Adjusted probability, % | - | - | - | - | - | - |
| **90 days** |  |  |  |  |  |  |
| ***≥1 return to the ED, n (%)*** | 106 (42.7) | 125 (50.4) |  | 83 (43.9) | 86 (41.8) |  |
| Adjusted probability, % | 34.4 [24.7-47.9] | 39.9 [28.4-56.1] | 0.14 | 37.3 [26.4-52.6] | 34.4 [24.9-47.4] | 0.48 |
| Adjusted ED LOS, days | 1.59 [1.01-2.49] | 0.94 [0.58-1.53] | **<0.01** | 1.47 [0.93-2.31] | 1.19 [0.77-1.84] | 0.13 |
| ***≥1 Rehospitalization, n (%)*** | 58 (23.4) | 87 (35.1) |  | 55 (29.1) | 60 (29.1) |  |
| Adjusted probability, % | 21.5 [13.5-34.2] | 33.0 [21.2-51.5] | **<0.01** | 27.8 [17.4-44.3] | 27.2 [17.8-41.6] | 0.90 |
| Adjusted hospital LOS, days | 16.92 [9.22-31.04] | 16.94 [9.29-30.87] | 0.99 | 8.41 [3.88-18.22] | 12.19 [6.40-23.21] | 0.20 |
| ***Death, n (%)*** | 14 (5.7) | 27 (10.9) |  | 10 (5.3) | 22 (10.7) |  |
| Adjusted probability, % | 6.1 [2.1-17.2] | 12.2 [4.6-32.1] | **0.03** | 4.8 [1.6-14.9] | 11.7 [4.8-28.7] | **0.03** |
| **180 days** |  |  |  |  |  |  |
| ***≥1 return to the ED, n (%)*** | 147 (59.3) | 159 (64.1) |  | 118 (62.4) | 109 (52.9) |  |
| Adjusted probability, % | 47.2 [37.2-59.9] | 50.0 [39.0-64.1] | 0.41 | 54.0 [42.4-68.7] | 44.1 [34.6-56.1] | **0.02** |
| Adjusted ED LOS, days | 1.11 [0.63-1.96] | 0.71 [0.38-1.33] | **0.01** | 1.01 [0.58-1.76] | 0.91 [0.53-1.54] | 0.52 |
| ***≥1 Rehospitalization, n (%)*** | 89 (35.9) | 124 (50.0) |  | 90 (47.6) | 80 (38.8) |  |
| Adjusted probability, % | 29.6 [20.8-42.2] | 38.8 [27.6-54.6] | **0.01** | 41.2 [29.2-58.1] | 32.6 [23.4-45.5] | **0.04** |
| Adjusted hospital LOS, days | 13.56 [7.29-25.19] | 15.13 [8.19-27.96] | 0.55 | 7.08 [3.26-15.37] | 10.64 [5.62-20.12] | 0.19 |
| ***Death, n (%)*** | 22 (8.9) | 44 (17.7) |  | 19 (10.1) | 33 (16.0) |  |
| Adjusted probability, % | 9.8 [4.7-20.3] | 21.8 [11.2-42.2] | **<0.01** | 11.9 [5.7-24.8] | 19.2 [10.4-35.4] | 0.08 |

CDA: Clinique des aînés; CI: Confidence Interval; ED : Emergency Department; SD: Standard Deviation

*Adjusted for propensity score, age, number of previous consultations and reason for consultation.
